# Enhancing diagnostic performance and image quality in coronary CT angiography: Impact of SnapShot Freeze 2 algorithm across varied heart rates in stent patients

**DOI:** 10.1002/acm2.14412

**Published:** 2024-05-28

**Authors:** Zhehao Wu, Qijia Han, Yuying Liang, Zhijuan Zheng, Minyi Wu, Zhu Ai, Kun Ma, Zhiming Xiang

**Affiliations:** ^1^ Postgraduate Cultivation Base of Guangzhou University of Chinese Medicine, Panyu Central Hospital Guangzhou China; ^2^ Department of Radiology Guangzhou Panyu Central Hospital Guangzhou China; ^3^ CT Imaging Research Center GE HealthCare China Guangzhou China

**Keywords:** computed tomography, coronary artery stent, motion correction

## Abstract

**Purpose:**

To investigate the enhancement of image quality achieved through the utilization of SnapShot Freeze 2 (SSF2), a comparison was made against the results obtained from the original SnapShot Freeze algorithm (SSF) and standard motion correction (STND) in stent patients undergoing coronary CT angiography (CCTA) across the entire range of heart rates.

**Materials and methods:**

A total of 118 patients who underwent CCTA, were retrospectively included in this study. Images of these patients were reconstructed using three different algorithms: SSF2, SSF, and STND. Objective assessments include signal‐to‐noise ratio (SNR), contrast‐to‐noise ratio (CNR), diameters of stents and artifact index (AI). The image quality was subjectively evaluated by two readers.

**Results:**

Compared with SSF and STND, SSF2 had similar or even higher quality in the parameters (AI, SNR, CNR, inner diameters) of coronary artery, stent, myocardium, MV (mitral valve), TV (tricuspid valve), AV (aorta valve), and PV (pulmonary valve), and aortic root (AO). Besides the above structures, SSF2 also demonstrated comparable or even higher subjective scores in atrial septum (AS), ventricular septum (VS), and pulmonary artery root (PA). Furthermore, the enhancement in image quality with SSF2 was significantly greater in the high heart rate group compared to the low heart rate group. Moreover, the improvement in both high and low heart rate groups was better in the SSF2 group compared to the SSF and STND group. Besides, when using the three algorithms, an effect of heart rate variability on stent image quality was not detected.

**Conclusion:**

Compared to SSF and STND, SSF2 can enhance the image quality of whole‐heart structures and mitigate artifacts of coronary stents. Furthermore, SSF2 has demonstrated a significant improvement in the image quality for patients with a heart rate equal to or higher than 85 bpm.

## 1 INTRODUCTION

Coronary computed tomography angiography (CCTA) is a widely used noninvasive imaging technique for diagnosing coronary artery disease (CAD). It is considered a simple, safe, and fast method, as evidenced, for example, by the guidelines set by the NICE, and the ESC.^1^, ^2^ CCTA plays a crucial role in detecting abnormalities in the structures of the heart, aorta, pulmonary arteries, heart valves, and coronary arteries.^3^, ^4^

CCTA has become increasingly important for evaluating in‐stent restenosis, and it might help to avoid invasive diagnostic procedures.^5^, ^6^, ^7^, ^8^, ^9^ However, the presence of metal artifacts caused by coronary artery stents often leads to a decline in image quality when assessing both the stents themselves and the surrounding structures.^10^, ^11^ Consequently, this affects the confidence of radiologists in making accurate diagnoses. Several factors contribute to the occurrence of metal artifacts on CCTA images, including beam hardening, scattering, noise, motion, and edge effects.^12^, ^13^

Several studies have emphasized that factors such as the diameter, type, and thickness of the implanted stent, contribute to non‐diagnostic CT image quality.^14^, ^15^, ^16^, ^17^ Importantly, high heart rate and heart rate variability (HRV) can result in motion artifacts and diminish image quality, making it a particularly significant contributing factor.^18^, ^19^

Currently, a motion correction algorithm known as the SSF (GE Healthcare) has been used for motion correction in coronary arteries. Recently, a second‐generation algorithm known as the SSF2 (GE Healthcare) has been proposed. The SSF utilizes information from adjacent cardiac phases captured during a single axial rotation to characterize motion at the desired target phase. On the other hand, the SSF2 algorithm, a fully automated technique based on knowledge and feedback obtained from SSF, seeks each region of all image volumes for a local path that is consistent with the subset of measured data. Once the vessel's motion path is identified, the data are discretized into a series of datasets based on when the corresponding projection rays were measured. Each volume dataset in the series undergoes the process of spatial deformation by the motion field. This allows the motion state to be mapped from the respective time to the central reference time that is determined by the prescribed cardiac phase.^20^, ^21^, ^22^ Therefore, the SSF2 extends its motion characterization and motion correction to the entire heart, which can not only further reduce coronary artery artifacts due to motion in patients with high heart rates but it also improves the image quality of other cardiac vascular structures such as valves and cardiac muscles on cardiovascular CT images.^23^

In a limited number of studies, SSF2 has demonstrated its potential to enhance the diagnostic accuracy of the three major coronary arteries and improve the image quality of non‐coronary regions (such as the MV, TV, AV, PV, myocardium, AS, VS, AO, and PA) in patients with high heart rate.^20^, ^23^ Additionally, the utilization of SSF2 has resulted in significant improvements in image quality in systolic data sets, enabling accurate measurement of aortic annular dimensions in patients undergoing pre‐transcatheter aortic valve replacement (TAVR).^21^, ^22^

Furthermore, in the field of artificial implants, research investigating the application of SSF2 to artificial valves has confirmed its ability to enhance the image quality of CT scans for mechanical valves compared to standard images. The notable reduction of motion artifacts achieved through the implementation of SSF2 may facilitate the improved detection of abnormalities in prosthetic valves.^24^

However, there is a noticeable lack of studies investigating the potential of SSF2 in enhancing the image quality of CCTA in patients with coronary artery stents. Consequently, we conducted a study wherein we collected CCTA images of patients with coronary artery stents. We then compared the image quality produced by three different algorithms (SSF2, SSF, and the uncorrected standard algorithm) to evaluate the efficacy of the two motion correction algorithms in rectifying motion artifacts in various anatomical structures, including the left anterior descending artery (LAD), left circumflex branch (LCX), right coronary artery (RCA), AO, left coronary artery (LCA), MV, TV, AV, PV, myocardium, AS, VS, AO, PA, and coronary artery stent. Additionally, we also assessed the disparities between SSF2 and SSF in improving the image quality of CCTA at both high and low heart rates.

## 2 MATERIALS AND METHODS

### 2.1 Study population

**FIGURE 1:**
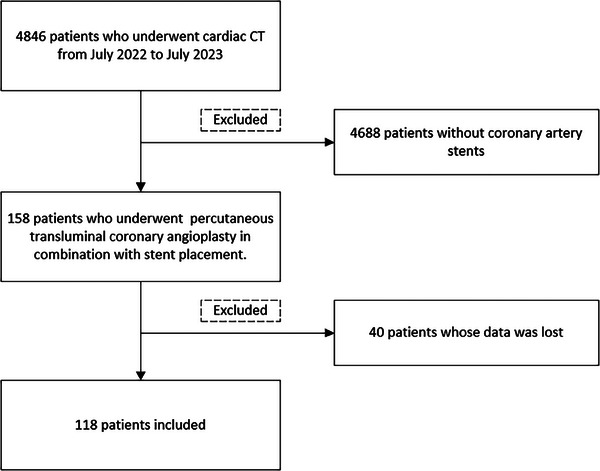
Flow chart of the study population.

This retrospective study, which aimed to investigate the effectiveness of SSF2 versus SSF in patients with previous stent implantation, was approved by the institutional review board of our hospital. Informed consent was waived for this study. We identified a cohort of 158 patients who were referred for CCTA and had undergone stent implantation prior to the procedure. This cohort was selected from a larger population of 1200 patients who underwent cardiac CT examinations between August 2022 and July 2023. A total of 1024 people without coronary stents were excluded. After excluding forty patients due to data loss that resulted in the inability to apply SSF2, a total of 118 patients with 177 stents were included in the final analysis (Figure 1).

### 2.2 Image acquisition

All CT examinations were conducted using a wide‐coverage, 256‐row detector (16 cm in z‐axis) CT scanner (Revolution Apex CT, GE HealthCare) with a tube rotation time of 280 ms. Prior to the examination, patients did not receive oral beta‐blockers for heart rate (HR) control.

The tube current was automatically adjusted between 100 and 1000 mA, and the noise index (NI) was set to be 24. A baseline kVp of 80 was selected for clinical purposes. All patients underwent scanning using the prospectively ECG‐triggered axial mode (Axial mode). The padding range for the R‐R interval was set at 0%−100% when a regular HR was recorded during pre‐exam monitoring. However, if the HRV exceeded 20 bpm, the padding range was adjusted to 0%−150%.

### 2.3 Image reconstruction

The automatic coronary artery analysis software of Advantage Workstation (AW 4.7, GE HealthCare) was utilized for the analysis. SmartPhase technology was employed to automatically select the reconstruction phases. Three groups of images were reconstructed: group A consisted of STND images, group B comprised SSF reconstruction images, and group C comprised SSF2 reconstruction images. Systolic and diastolic images were obtained for each of the three groups.

### 2.4 Objective evaluation

One radiologist with over 3 years of working experience in CCTA diagnosis evaluated the objective image quality and delineated a region of interest (ROI). Due to individual differences in coronary arteries and coronary stents in different patients, ROI sizes vary slightly from patient to patient. The goal was to keep the size of each ROI at 5−10 mm^2^, so ROI size did not vary greatly from patient to patient. Secondly, our evaluation is a comparison of the three algorithms, and the ROI of the same patient in the reconstructed images of the three algorithms was propagated through the GE post‐processing workstation (AW4.7), which made the ROI of each patient consistent in size and position. Thus, so even if the ROI size of different patients was different, it would not affect the comparison between the three algorithms.

The images of all cases were reconstructed using SSF2 and SSF techniques, while the standard (STND) images were retained. The CT number and SD values were obtained from each ROI including the AO, LCA, LAD, RCA, myocardium, AV, and PV. Additionally, the SNR and CNR were calculated for all of these tissues.

We outlined the ROIs at the center of the stent (in‐stent) to acquire the CT value and SD value, as well as the CT value of the ROI in subcutaneous adipose tissue near the aortic root, and inner diameters of stents were measured. Then, we calculated the AI and CNR according to the following formulae^25^ (AI is a value that reflects the impact of artifacts on image quality. The higher the value, the greater the impact of artifacts on the image and the lower the image quality.) Myocardial attenuation and noise were used as a reference. The selection of the ROI aimed to avoid calcified plaques as much as possible and opted for areas with more uniform density.

The formulae of SNR and CNR of coronary arteries, valves, myocardium; CNR and AI of stents were as follows:

**Calculation formulae**:

(1) $${\text{SNR}}_{lumen}=CT_{lumen}/SD_{lumen}$$

(2) $${\text{CNR}}_{lumen}=\left(CT_{lumen}-CT_{fat}\right)/SD_{fat}$$

(3) $$AI=\sqrt{{\left(SD_{artifact}\right)}^{2}-{\left(SD_{myo\;cardium}\right)}^{2}};$$

(4) $$\begin{array}{ccc} & & {\text{CNR}}_{artifact}=/CT_{artifact}-CT_{myo\;cardium}|\\ & & /\sqrt{{\left(CT_{artifact}\right)}^{2}-{\left(CT_{myo\;cardium}\right)}^{2}} \end{array}$$

### 2.5 Subjective evaluation

All images were assessed by two radiologists each with 3 years of experience in cardiovascular radiology, who were blinded to the clinical information and the reconstruction algorithm used for these images. In cases where there were discrepancies in the scores assigned by the two readers, a third radiologist was consulted to determine the final score.

#### 2.5.1 Coronary artery subjective score

A 5‐point scale was used to evaluate coronary artery based on the presence of motion artifacts. A score of 1 indicates very poor, non‐evaluable; a score of 2 indicates poor, severe motion artifacts and non‐evaluable; a score of 3 indicates adequate, moderate artifacts that are clinically diagnosable; a score of 4 indicates good, minor artifacts; and a score of 5 indicates excellent, with no motion artifacts and clear delineation of the segment. Scores of 3−5 were considered diagnostic, while scores of 4−5 were considered excellent. The coronary image quality of the three algorithms were compared.^26^

#### 2.5.2 Subjective assessment of stent image quality

The image quality of the stents was evaluated by observers using a four‐point grading scale. A score of 1 indicated that the stent appeared as a clear area without any motion artifacts. A score of 2 indicated the presence of discrete blurring and small streak artifacts in the stent image. A score of 3 indicated blurred stent margins and moderate broader streak artifacts. A score of 4 indicated inadequate differentiation between the stent and its surrounding tissue. In the assessment of coronary stent image quality, a score of 4 indicated that the stent was deemed unacceptable.^27^, ^28^

#### 2.5.3 Subjective assessment of other structures

The image quality of AO, PA, AS, VS, MV, TV, AV, PV was evaluated using a 4‐point grading scale system,^29^ The evaluation criteria included the presence of motion artifacts, delineation between the object and surrounding tissue, and interpretability for quantitative measurements. A score of 1 indicated a nondiagnostic image with severe motion artifacts and inadequate differentiation between the object and surrounding tissue. A score of 2 indicated a detectable image with moderate motion artifacts and noticeably blurred margins, which was acceptable for detection but not suitable for quantitative measurement. A score of 3 indicated a measurable image with minor motion artifacts and somewhat blurred margins, allowing for full evaluation. A score of 4 indicated an excellent image with no motion artifacts present. Cardiac structures with scores greater than 1 were considered interpretable.

#### 2.5.4 Correlation between subjective score of stents and HRV

We divided the subjective scores of LAD stent, RCA stent and LCX stent image quality of different algorithms into two groups (1 point is divided into the first group; 2,3, and 4 points is divided into the second group), and evaluated the correlation between subjective image quality scores and HRV.

### 2.6 Evaluation of image quality improvement at different heart rates

These patients were categorized into two groups based on their heart rate: a high heart rate group (>85 beats/min) and a low heart rate group (≤85 beats/min). We choose 85 beats/min as a cutoff base on previous research evidence.^30^, ^31^ The impact of CCTA image with SSF2 reconstruction was evaluated and compared to SSF reconstruction images and STND reconstruction images in terms of both subjective and objective measurements. Firstly, we calculated the improvement ratios for all anatomical locations, including the vessels (AO, LCA, LAD, RCA), stents (LM stent, LAD stent, LCX stent, RCA stent), valves (MV, TV, AV, PV), and myocardium (specific values are detailed in Tables S4–S11). Secondly, we computed the average improvement ratios for these four segments separately. Since they belong to the same type of data, we aggregated the data from each segment to calculate the average improvement ratios, thereby combining multiple scores into a single growth rate.

This evaluation was conducted in order to assess the effectiveness of SSF2 reconstruction in improving image quality for patients with varying heart rates. The ratio of improvement, as compared to the STND algorithm, was used for the assessment, which shows the average growth rate of using SSF2 and SSF in different locations. Ratio of SSF2 improvement = (SSF2 value—STND value)/STND value; ratio of SSF improvement = (SSF value—STND value)/STND value.

### 2.7 Statistical analysis

SPSS 26.0 statistical analysis software was utilized for data analysis. The patient's age followed a normal distribution and was presented as mean ± standard deviation (x ± s). In order to evaluate the objective image quality of SSF2, SSF, and STND, paired sample *t*‐tests were employed for data that conformed to a normal distribution, while the nonparametric test Wilcoxon signed rank test was used for data that exhibited evidence of non‐normality. The chi‐square test was employed to compare the subjective evaluation results of SSF2, SSF, and STND. To minimize the probability of type I errors in pairwise comparisons, the Bonferroni correction was utilized. Furthermore, in cases where subjective and objective comparisons were made three times, the test level was set at a = 0.05/3 = 0.0167. Therefore, a statistically significant difference was considered when *p* < 0.0167. Correlation between subjective score of stents and HRV using two‐sample rank test, *p* < 0.05 is statistically significant. The consistency of image quality scores among observers was evaluated using weighted kappa statistics.

## 3 RESULTS

### 3.1 Study population characteristics

The average age of the patients was 63.7 ± 11.7 years (ranging from 32.0 to 87.0 years), consisting of 87 males (74%), and 31 females (26%). Among the patients, 27 had a high heart rate (≥85 bpm), while 91 had a low heart rate. Table 1 presents the characteristics of the study population, including the radiation doses for CCTA and CT scan parameters.

**TABLE 1:** Study characteristics of participants and CCTA scanning parameters, radiation dose.

Patient's characteristics	
**Mean age (years)**	63.7 ± 11.7 (32–87)
**Sex**	
Male	87(74%)
Female	31(26%)
**Heart rate (beats per min)**	74.1 ± 13.9 (52–123)
≥85 bpm	27 (21%)
<85 bpm	91 (79%)
**Stent location**	
Left main coronary artery	89 (50%)
Left anterior descending coronary artery	35 (20%)
Left circumflex coronary artery	46 (26%)
Right coronary artery	7 (4%)
**Heart rate during scan acquisition (beats/min)**	71.1 ± 12.8
**Heart rate variability (beats/min)**	10.7 ± 10.2
Tube voltage (kVp)	80
Tube current (mA)	100–1000
CTDIvol (mGy)	15.2 ± 7.6 (3.64–58.87)
DLP (mGy/cm)	221.7 ± 113.6 (59.79–824.21)

*Note*: Values are represented as mean ± SD (range) or *n* (%).

Abbreviations: CCTA, coronary computed tomography angiography; kVp, kilovolt Peak; mA, milliampere.

### 3.2 Stent location

Among these stents, there were 89 in the left anterior descending artery (LAD), 35 in the left circumflex artery (LCX), 46 in the right coronary artery (RCA), and 7 in the left main artery (LM).

### 3.3 Image quality analysis

In terms of coronary artery image quality, the objective measurements of the SSF2 group were superior to those of the SSF group, and there were significant differences between the two groups (all *p* < 0.0167). When compared to the STND group, the SSF2 group exhibited significantly higher AO, LAD, LCA, RCA, MV, TV SNR and LCA, CNR at systolic and diastolic phases, as well as higher myocardium, AV SNR at diastolic phase, LAD, CNR at systolic phase (Tables 2 and S1).

**TABLE 2:** Objective assessment of arteries, myocardium and MV, TV, AV, PV.

Parameters	Locations	Period	SSF2 Mean ± SD	SSF Mean ± SD	STD Mean ± SD
SNR *n* = 118	AO	Systole	31.19 ± 8.03	30.78 ± 8.06	30.75 ± 8.40
		Diastole	32.19 ± 8.28	31.72 ± 8.29	31.58 ± 8.59
	LCA	Systole	20.24 ± 7.47	18.29 ± 7.82	18.37 ± 8.18
		Diastole	21.85 ± 9.71	18.95 ± 8.51	19.17 ± 9.07
	LAD	Systole	14.94 ± 10.02	12.61 ± 5.53	12.55 ± 5.71
		Diastole	13.55 ± 5.03	12.12 ± 5.18	12.03 ± 5.45
	RCA	Systole	17.06 ± 7.59	15.49 ± 7.27	14.81 ± 7.36
		Diastole	17.45 ± 8.84	14.46 ± 6.42	14.68 ± 6.85
	Myocardium	Systole	4.73 ± 1.49	4.50 ± 1.46	4.64 ± 1.52
		Diastole	4.68 ± 1.26	4.49 ± 1.24	4.52 ± 1.30
	Mitral valve	Systole	18.07 ± 6.24	16.91 ± 5.79	17.08 ± 5.85
		Diastole	20.39 ± 6.43	19.54 ± 6.09	19.73 ± 6.26
	Tricuspid valve	Systole	5.12 ± 2.58	4.87 ± 2.44	4.91 ± 2.52
		Diastole	4.78 ± 2.18	4.65 ± 2.09	4.69 ± 2.20
	Pulmonary valve	Systole	9.83 ± 4.89	9.63 ± 4.87	9.79 ± 4.90
		Diastole	11.41 ± 6.46	11.18 ± 6.19	11.21 ± 6.26
	Aortic valve	Systole	23.06 ± 6.39	21.75 ± 6.42	22.59 ± 6.42
		Diastole	25.80 ± 6.63	24.87 ± 6.52	25.17 ± 6.70
CNR *n* = 118	AO	Systole	37.29 ± 11.91	36.10 ± 12.42	36.49 ± 12.95
		Diastole	38.07 ± 12.46	36.56 ± 12.01	37.63 ± 12.83
	LCA	Systole	33.84 ± 9.99	31.92 ± 9.21	32.71 ± 9.61
		Diastole	32.86 ± 9.05	31.16 ± 9.13	31.02 ± 9.64
	LAD	Systole	31.88 ± 15.59	29.44 ± 11.2	29.31 ± 11.38
		Diastole	31.41 ± 10.93	29.90 ± 10.98	30.40 ± 11.64
	RCA	Systole	33.17 ± 11.35	31.88 ± 11.62	32.28 ± 12.26
		Diastole	33.44 ± 11.59	31.49 ± 11.47	32.29 ± 12.09
	Myocardium	Systole	9.35 ± 2.99	9.13 ± 2.96	9.24 ± 3.15
		Diastole	9.94 ± 3.16	9.55 ± 3.06	9.84 ± 3.32
	Mitral valve	Systole	35.27 ± 11.51	34.11 ± 12.01	34.49 ± 12.53
		Diastole	35.85 ± 12.15	34.37 ± 11.52	35.38 ± 12.31
	Tricuspid valve	Systole	12.59 ± 7.24	12.16 ± 7.04	12.23 ± 6.94
		Diastole	12.63 ± 7.09	12.03 ± 6.37	12.37 ± 6.62
	Pulmonary valve	Systole	15.18 ± 8.05	14.80 ± 8.07	14.92 ± 8.13
		Diastole	16.26 ± 8.18	15.57 ± 7.21	15.60 ± 7.93
	Aortic valve	Systole	36.65 ± 11.64	35.23 ± 12.38	35.81 ± 12.59
		Diastole	37.26 ± 12.19	35.93 ± 11.77	36.79 ± 12.94

*Note*: Data are mean ± standard deviations.

Abbreviations: AO, aorta; CNR, contrast to noise ratio; LAD, left anterior descending artery; LCA, left coronary artery; RCA, right coronary artery; SNR, signal‐to‐noise ratio.

Regarding to the image quality of coronary artery stents, the systolic and diastolic AI values of the LAD, LCX, and RCA in the SSF2 group showed a notable decrease, indicating reduced artifacts compared to the SSF and STND groups. Additionally, the CNR of the LAD, LCX, and RCA in the SSF2 group exhibited a significant increase, further confirming improved image quality. Moreover, the stent inner diameter of the LAD experienced a considerable increase as well (all *p* < 0.0167, as shown in Tables 3 and S2).

**TABLE 3:** Objective assessment of stents.

Parameter	Location	Period	SSF2 Mean ± SD	SSF Mean ± SD	STD Mean ± SD
AI	LAD stent *n* = 89	Systole	53.49 ± 22.73	69.46 ± 36.11	73.01 ± 35.28
		Diastole	52.47 ± 20.46	64.44 ± 32.04	65.16 ± 31.14
	LCX stent *n* = 35	Systole	57.33 ± 21.92	77.04 ± 40.15	83.55 ± 43.62
		Diastole	55.34 ± 20.58	69.33 ± 30.91	71.42 ± 25.15
	RCA stent *n* = 46	Systole	52.50 ± 17.73	59.99 ± 26.02	71.06 ± 33.64
		Diastole	55.56 ± 18.28	73.23 ± 52.42	71.31 ± 42.82
	LM stent *n* = 7	Systole	31.67 ± 15.81	46.29 ± 20.96	47.88 ± 16.17
		Diastole	41.40 ± 12.10	57.24 ± 23.72	59.93 ± 14.97
CNR of stents	LAD stent *n* = 89	Systole	14.49 ± 5.15	13.17 ± 5.40	12.76 ± 5.27
		Diastole	15.01 ± 5.09	13.86 ± 5.45	13.82 ± 5.63
	LCX stent *n* = 35	Systole	16.09 ± 4.76	13.59 ± 4.46	12.75 ± 4.87
		Diastole	15.45 ± 5.61	13.94 ± 6.09	13.27 ± 5.51
	RCA stent *n* = 46	Systole	15.65 ± 5.25	14.41 ± 4.70	13.31 ± 4.60
		Diastole	15.09 ± 5.72	13.37 ± 5.77	13.09 ± 5.04
	LM stent *n* = 7	Systole	21.14 ± 6.39	16.91 ± 5.82	16.56 ± 5.41
		Diastole	18.01 ± 7.59	15.16 ± 8.21	13.95 ± 7.05
Inner diameters	LAD stent *n* = 89	Systole	1.48 ± 0.48	1.321 ± 0.52	1.3 ± 0.56
		Diastole	1.42 ± 0.45	1.33 ± 0.48	1.30 ± 0.50
	LCX stent *n* = 35	Systole	1.28 ± 0.46	1.24 ± 0.48	1.18 ± 0.49
		Diastole	1.3 ± 0.46	1.24 ± 0.47	1.24 ± 0.47
	RCA stent *n* = 46	Systole	1.60 ± 0.52	1.54 ± 0.51	1.43 ± 0.54
		Diastole	1.57 ± 0.64	1.52 ± 0.64	1.39 ± 0.59
	LM stent *n* = 7	Systole	1.96 ± 0.65	1.89 ± 0.66	1.89 ± 0.72
		Diastole	1.86 ± 0.48	1.71 ± 0.60	1.77 ± 0.73

*Note*: Data are mean ± standard deviations.

Abbreviations: AI, Artifact index; CNR, contrast to noise ratio; LAD, left anterior descending artery; LCX, Left circumflex branch; LM, left main artery; RCA, right coronary artery.

**FIGURE 2:**
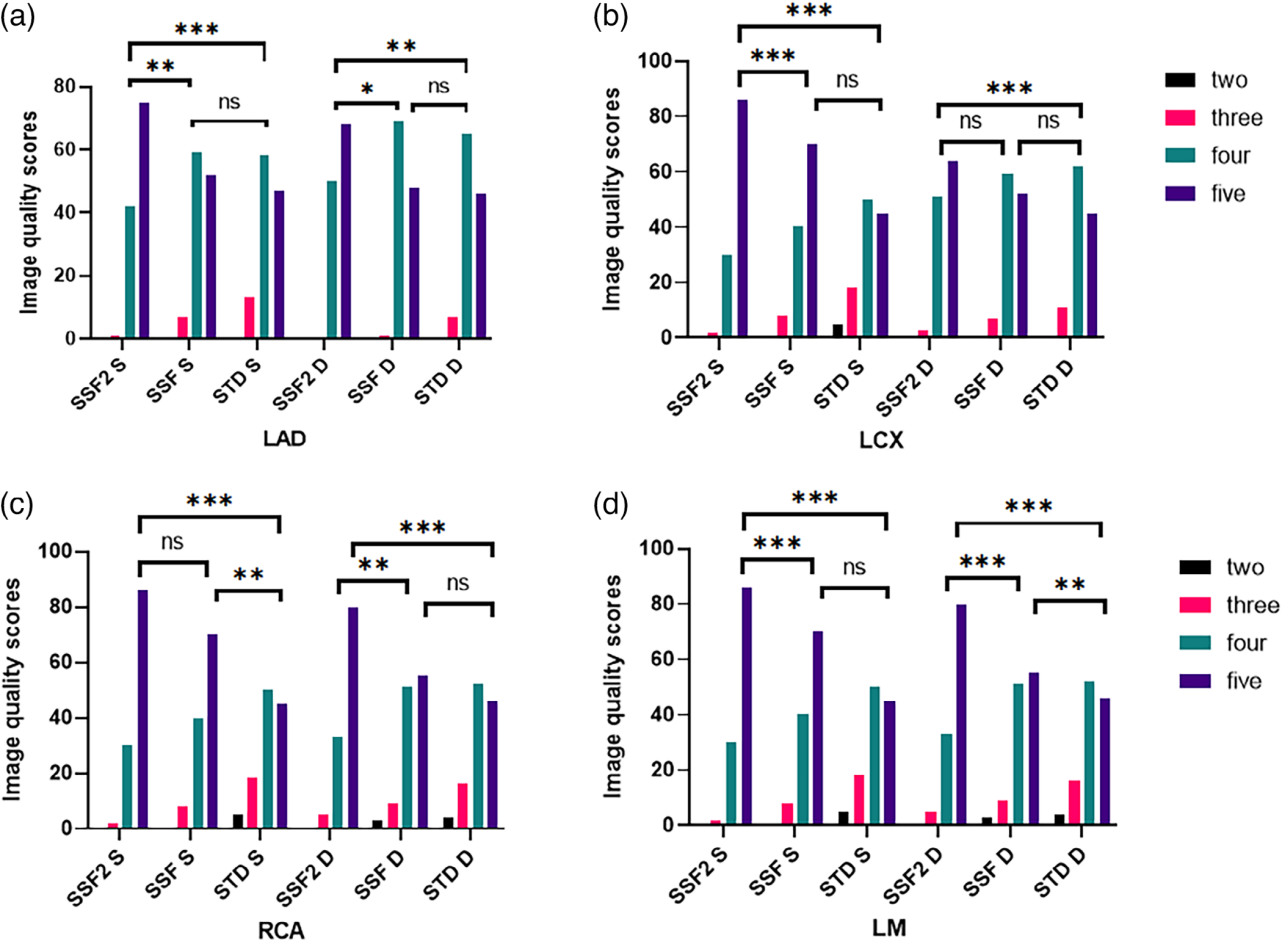
Subjective evaluation of coronary artery (LAD, left anterior descending artery; LCX, Left circumflex branch; RCA, right coronary artery; LM, left main artery; S, systole; D, diastole; The asterisks *, **, and *** represent statistical significance levels of *p* < 0.0167, <0.0033, and <0.0003, respectively, ns stands for no statistical significance).

Furthermore, when compared to the SSF group, the subjective scores of LM, LAD for systolic and diastolic phases, subjective score of LCX for systolic phase, and subjective score of RCA for diastolic phase were significantly higher in the SSF2 group, with the differences being statistically significant (all *p* < 0.0167) (Figure 2). The value of weighted kappa between the two radiologists was very high, ranging from 0.79 to 0.884.

Furthermore, subjective evaluations revealed that the SSF2 group achieved higher subjective scores of the LAD stent for both the systolic and diastolic phases compared to the SSF group (Figure S1).

**FIGURE 3:**
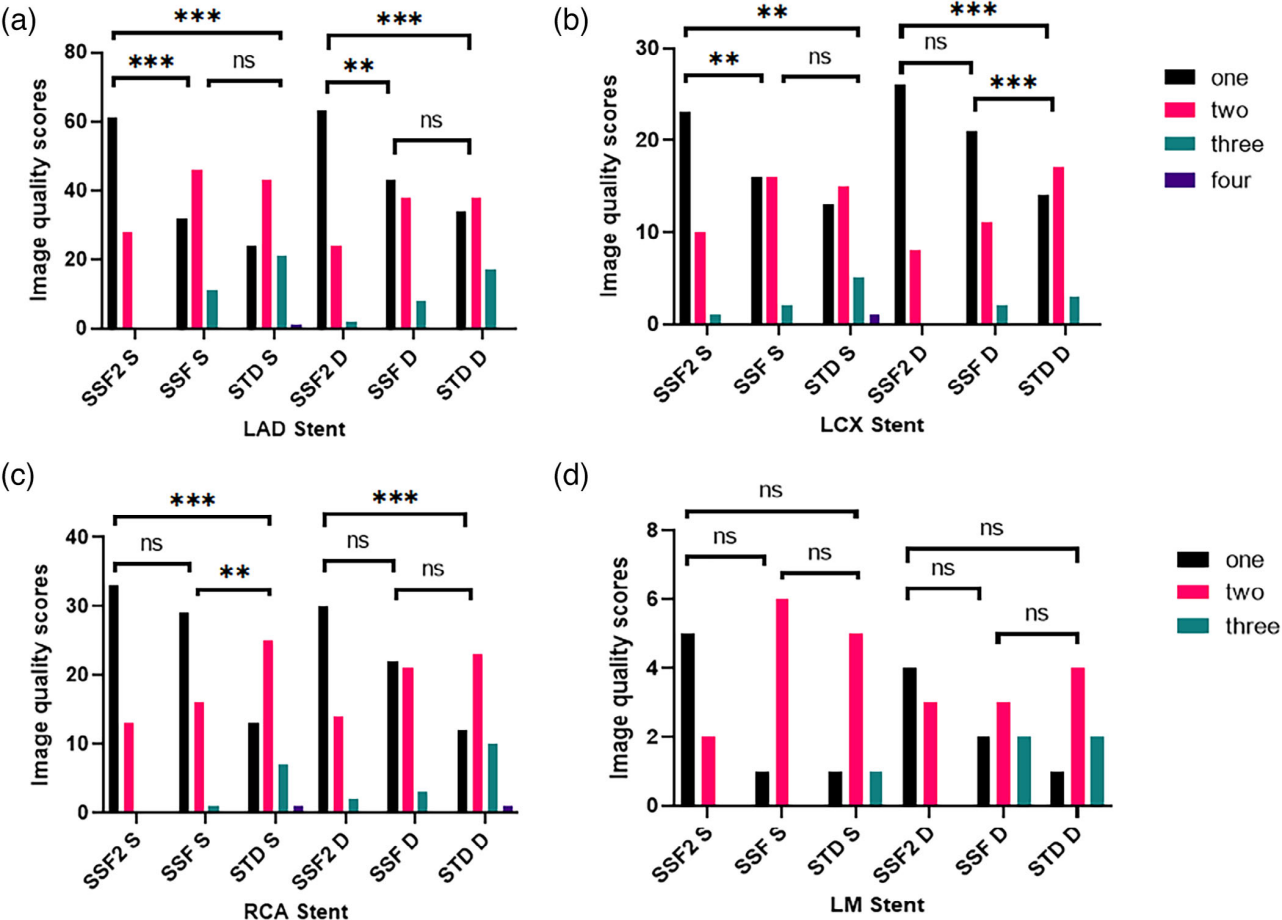
Subjective evaluation of coronary stent (One, two, three, four represent subjective evaluation scores. LAD, left anterior descending artery; LCX, Left circumflex branch; RCA, right coronary artery; LM, left main artery; S, systole; D, diastole; The asterisks *, **, and *** represent statistical significance levels of *p* < 0.0167, <0.0033 and, <0.0003, respectively, ns stands for no statistical significance).

During the systolic phase, the subjective score of the LCX stent was notably higher in the SSF2 group than in the SSF group. However, there was no significant difference in subjective scores of the RCA stent and LM stent between the SSF2 group and SSF group during systolic and diastolic periods (Figure 3). The value of weighted kappa between the two radiologists was exceptionally high, ranging from 0.755 to 1. On the other hand, no significant differences were observed in other subjective scores.

**FIGURE 4:**
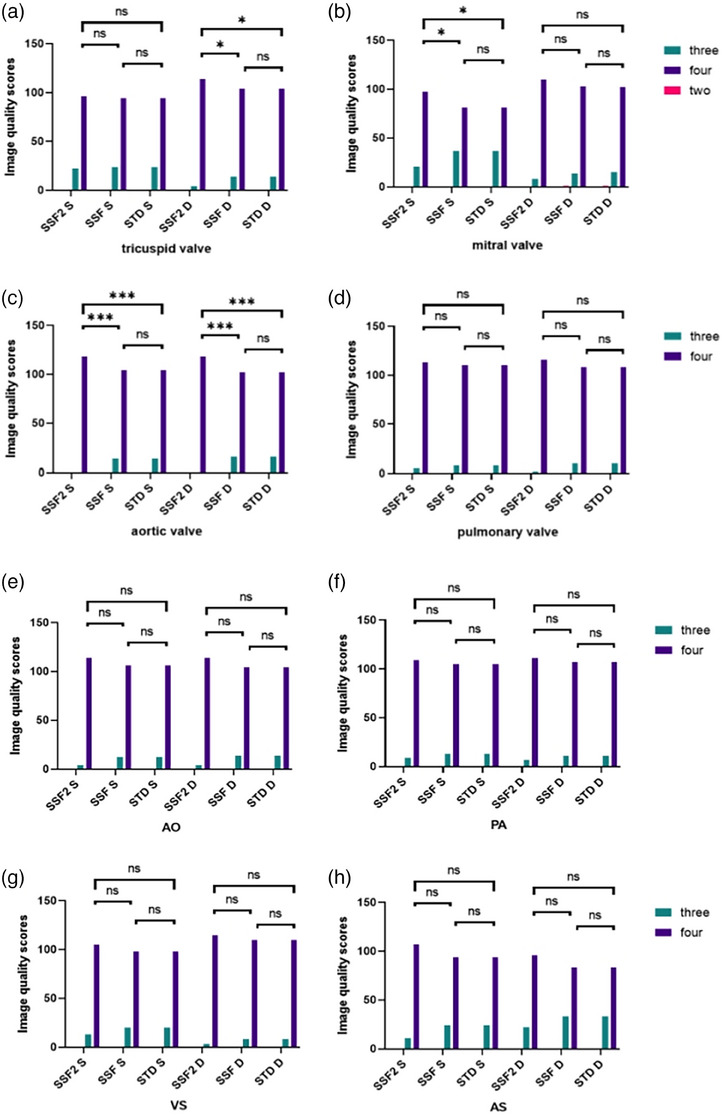
Subjective evaluation of AO, PA, AS, VS, MV, TV, AV, PV (AO, aorta root; PA, pulmonary artery root; AS, Atrial septum; VS, ventricular septum; MV, mitral valve; TV, tricuspid valve; AV, aortic valve; PV, pulmonary valve; S, systole; D, diastole; The asterisks *, **, and *** represent statistical significance levels of *p* < 0.0167, <0.0033, and <0.0003, respectively, ns stands for no statistical significance.

In the subjective scores of the area outside the coronary artery and stent, significant increases were observed in the systolic and diastolic of AO, as well as the systolic score of MV and the diastolic score of TV in the SSF2 group, when compared to both the SSF group and STND group (all *p* < 0.0167) (Figures 4 and S2). The value of weighted kappa between the two radiologists was found to be high, ranging from 0.679 to 0.967.

Subjective stent scores in systolic and diastolic phases for the three algorithms had little correlation with heart rate variability (all *p*>0.05), except for RCA diastolic STND images(*p* = 0.033) (Table S3).

### 3.4 Effect of SSF2 and SSF on CCTA image quality with different heart rate

Compared to the STND image, the SSF2 image exhibited a remarkable enhancement in terms of image quality, as evidenced by the subjective and objective index scores of CCTA coronary artery, coronary artery stent, MV, TV, AV, PV, and myocardium in the high heart rate group. Notably, the improvement ratios of SSF2 relative to STND were larger in the high heart‐rate group than in the low heart‐rate group. The improvement ratios relative to STND were larger in the SSF2 group than in the SSF group.

**FIGURE 5:**
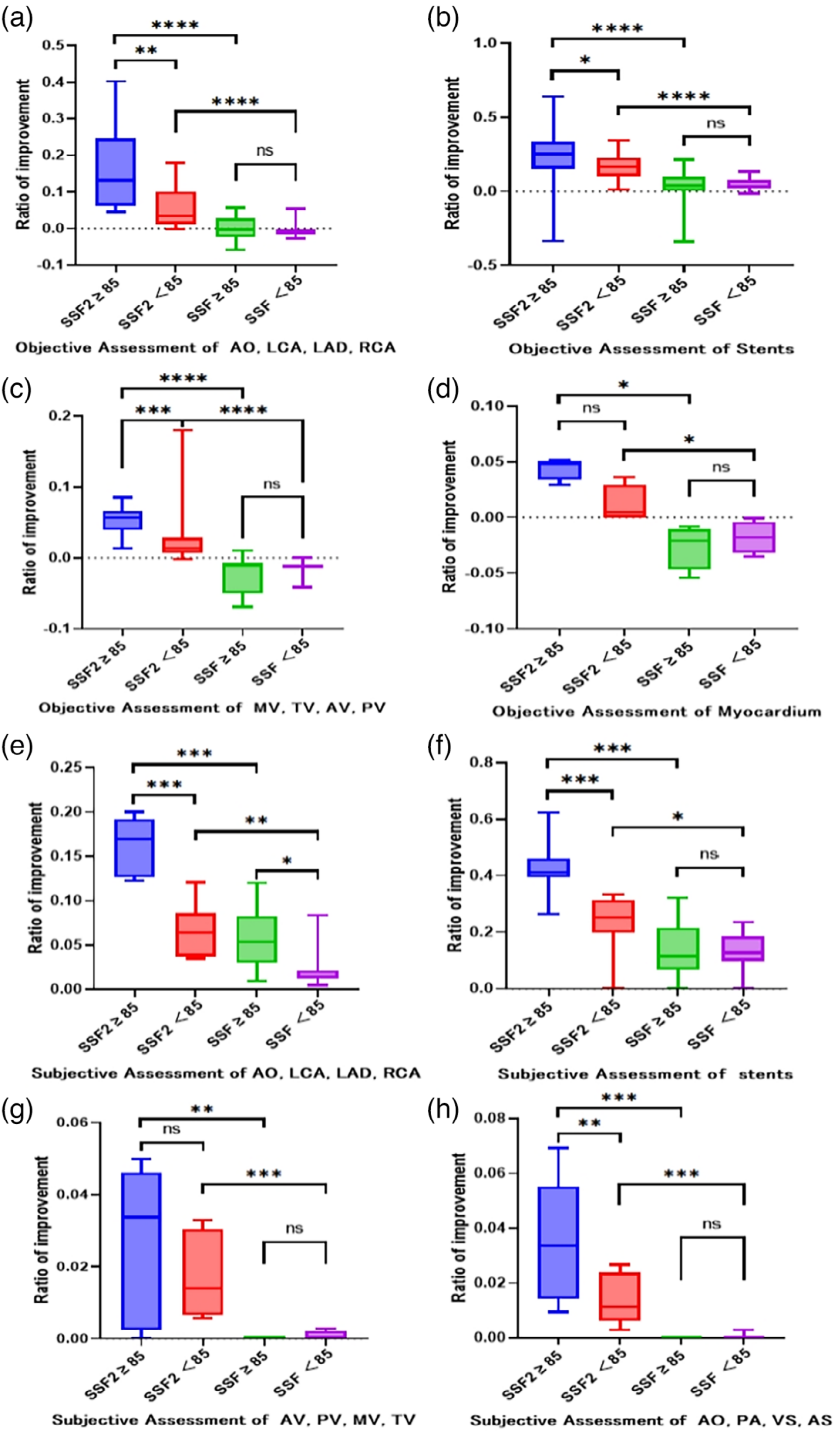
Effect of SSF2 and SSF on CCTA Image quality with different heart rate (Figure 5 shows the average growth rate of using SSF2 and SSF in different locations. The specific growth rate is shown in the appendix). (a) Objective assessment of arteries. Made up of the growth rate of SNR and CNR. (b) Objective assessment of Stents. Made up of the growth rate of AI, CNR, and Diameter of stent. (c) Objective assessment valves. Made up of the growth rate of SNR and CNR. (d) Objective assessment myocardium. Made up of the growth rate of SNR and CNR. (e) Subjective assessment of arteries. Made up of the growth rate of subjective scores. (F) Subjective assessment stents. Made up of the growth rate of subjective scores. (g) Subjective assessment of valves. Made up of the growth rate of subjective scores. (h) Subjective assessment AS, VS, AO, PA. Made up of the growth rate of subjective scores. (The asterisks *, **, and *** represent statistical significance levels of *p* < 0.0167, <0.0033, and <0.0003, respectively, ns stands for no statistical significance.).

The objective evaluation of AO, LCA, LAD, and RCA consisted of improvement ratios of SNR and CNR (Presented in Table S4 and Figure 5a). There was a respective change of 15.7%, 5.8%, −0.1%, and −0.7% observed in patients belonging to the SSF2 high heart rate group, SSF2 low heart rate group, SSF high heart rate group, and SSF low heart rate group. Statistically significant differences were observed between the high and low heart rate group in SSF2, as well as between the SSF2 high heart rate group and the SSF high heart rate group, and between the SSF2 low heart rate group and the SSF low heart rate group. However, no significant difference was found between the SSF high heart rate group and the SSF low heart rate group.

The objective evaluation of stents consisted of improvement ratios of AI, CNR and Diameter of stent (Presented in Table S5 and Figure 5b). There was a respective increase of 22.5%, 16.9%, 3.1%, and 4.4% observed in patients belonging to the SSF2 high heart rate group, SSF2 low heart rate group, SSF high heart rate group, and SSF low heart rate group. Statistical analysis revealed significant differences between the SSF2 high heart rate group and SSF2 low heart rate group, as well as between the SSF2 high heart rate group and SSF high heart rate group, and between the SSF2 low heart rate group and SSF low heart rate group. However, no significant difference was found between the SSF high heart rate group and SSF low heart rate group.

The objective evaluation of MV, TV, AV, PV consisted of improvement ratios of SNR and CNR (Presented in Table S6 and Figure 5c). There was a respective change of 5.3%, 2.7%, −2.2%, and −1.3% observed in patients belonging to the SSF2 high heart rate group, SSF2 low heart rate group, SSF high heart rate group, and SSF low heart rate group. Statistically significant differences were observed between the high and low heart rate group in SSF2, as well as between the SSF2 high heart rate group and the SSF high heart rate group, and between the SSF2 low heart rate group and the SSF low heart rate group. However, no significant difference was found between the SSF high heart rate group and the SSF low heart rate group.

The objective evaluation of myocardium consisted of improvement ratios of SNR and CNR (Presented in Table S7 and Figure 5d). There was a respective change of 4.4%, 1.1%, −2.6%, and −1.8% observed in patients belonging to the SSF2 high heart rate group, SSF2 low heart rate group, SSF high heart rate group, and SSF low heart rate group. Statistically significant differences were observed between the SSF2 high heart rate group and the SSF high heart rate group, as well as the SSF2 low heart rate group and the SSF low heart rate group. However, no significant difference was found between the high and low heart rate group in SSF2, also no significant between the SSF high heart rate group and the SSF low heart rate group.

The subjective assessments of AO, LCA, LAD, and RCA consisted of improvement ratios of subjective scores (Presented in Table S8 and Figure 5e). There was a respective increase of 16.2%, 6.7%, 5.7%, and 2.3% observed in patients belonging to the SSF2 high heart rate group, SSF2 low heart rate group, SSF high heart rate group, and SSF low heart rate group. Statistically significant differences were observed between the high and low heart rate group in SSF2, as well as between the SSF2 high heart rate group and the SSF high heart rate group, and between the SSF2 low heart rate group and the SSF low heart rate group, and between the SSF high heart rate group and the SSF low heart rate group.

The subjective assessments of stents consisted of improvement ratios of subjective scores (Presented in Table S9 and Figure 5f). There was a respective increase of 42.7%, 23.3%, 14%, and 13.3% observed in patients belonging to the SSF2 high heart rate group, SSF2 low heart rate group, SSF high heart rate group, and SSF low heart rate group. Statistically significant differences were observed between the high and low heart rate group in SSF2, as well as between the SSF2 high heart rate group and the SSF high heart rate group, and between the SSF2 low heart rate group and the SSF low heart rate group. However, no significant difference was found between the SSF high heart rate group and the SSF low heart rate group.

The subjective assessments of MV, TV, AV and PV consisted of improvement ratios of subjective scores (Presented in Table S10 and Figure 5g). There was a respective increase of 2.7%, 1.8%, 0%, and 0.1% observed in patients belonging to the SSF2 high heart rate group, SSF2 low heart rate group, SSF high heart rate group, and SSF low heart rate group. Statistically significant differences were observed between the SSF2 high heart rate group and the SSF high heart rate group, as well as the SSF2 low heart rate group and the SSF low heart rate group. However, no significant difference was found between the high and low heart rate group in SSF2, also no significant between the SSF high heart rate group and the SSF low heart rate group.

The subjective assessments of AS, VS, AO, and PA consisted of improvement ratios of subjective scores (Presented in Table S11 and Figure 5h). There was a respective increase of 3.6%, 1.4%, 0%, and 0% observed in patients belonging to the SSF2 high heart rate group, SSF2 low heart rate group, SSF high heart rate group, and SSF low heart rate group. Statistically significant differences were observed between the high and low heart rate group in SSF2, as well as between the SSF2 high heart rate group and the SSF high heart rate group, and between the SSF2 low heart rate group and the SSF low heart rate group. However, no significant difference was found between the SSF high heart rate group and the SSF low heart rate group.

## 4 DISCUSSION

In this study, we aimed to assess the effectiveness of SSF2 and SSF, in correcting cardiac structure and motion artifacts of coronary stents by reconstructing cardiac images of patients with different heart rates. Our findings demonstrated that SSF2 enhanced the subjective and objective image quality in terms of coronary artery, myocardium, MV, TV, AV, PV, and stent, compared to SSF. When using SSF2, the improvement in image quality was more pronounced in the high heart‐rate group. However, there was no significant difference in image quality improvement between high heart rate stent patients and low heart rate stent patients when using SSF. Besides, when using the three algorithms, heart rate variability basically has no significant effect on stent image quality.

In our study, we observed that SSF2 not only improved image quality of the coronary artery and myocardium, but also partially improved the image quality of the MV, TV, AV, and PV. The improvement achieved with SSF2 was found to be superior to that of the SSF, consistent with previous research findings.^20^, ^23^ However, it is worth noting that the impact of SSF2 on non‐coronary areas was not as satisfactory, which may be attributed to the generally lower heart rate observed in our patient population.

**FIGURE 6:**
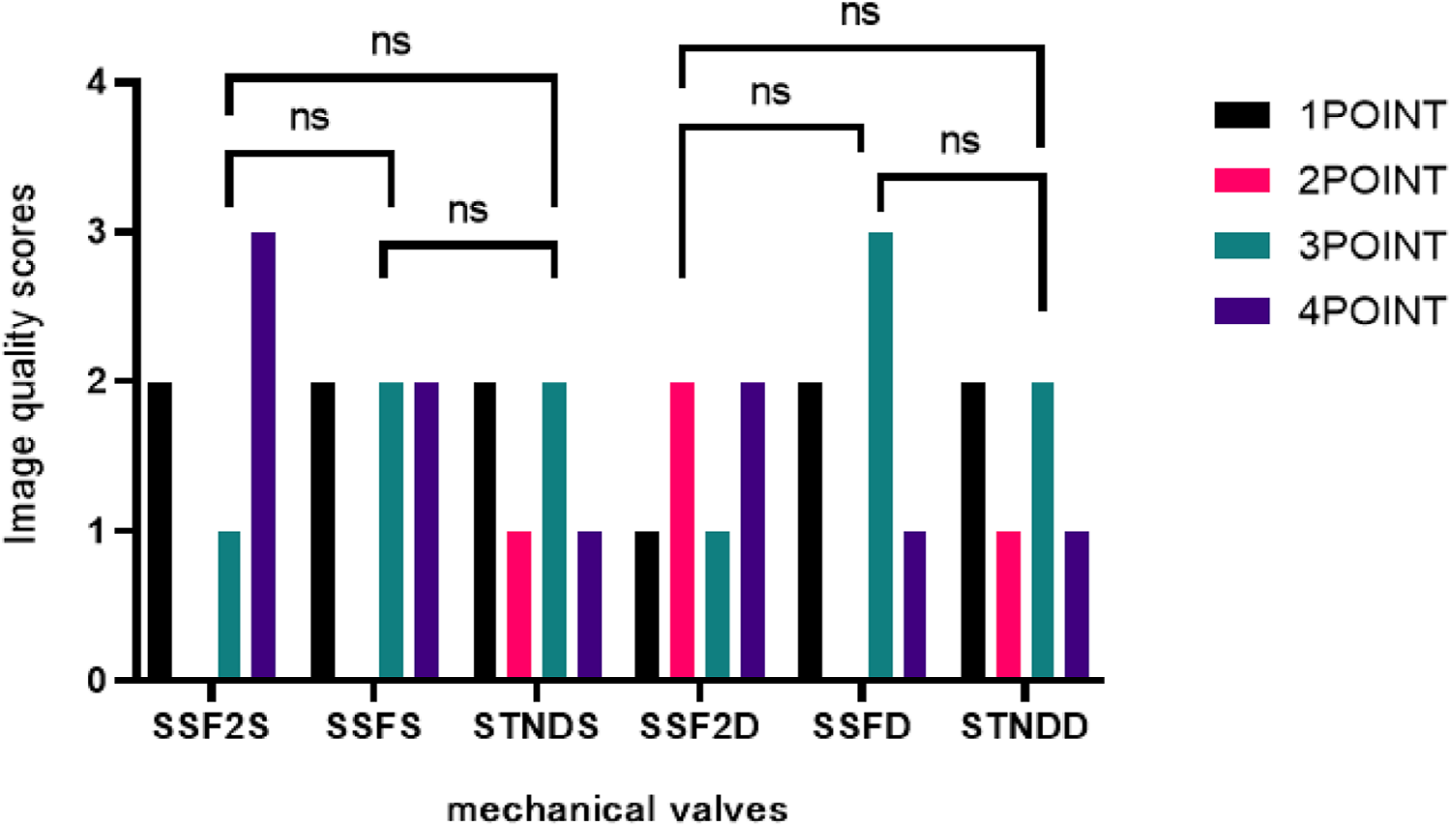
Subjective evaluation of mechanical valves (NS stands for no statistical significance).

In previous study, the effectiveness of the SSF2 in enhancing the image quality of CT scans of mechanical valves in patients was demonstrated when compared to STND images.^24^ In our study (consisting of data from 6 cases involving 2 aortic valves and 4 mitral valves), the artificial valve region of the SSF2, SSF, and STND images were subjected to a subjective evaluation (Figure 6). The mean and standard deviation of subjective score of SSF2 (2.75 ± 1.29) are better than those of SSF (2.58 ± 1.24) and STND (2.33 ± 1.15), which is consistent with the results of previous studies.

Besides, we found that the application of SSF2 improved the image quality of CCTA in patients with stents, which has not been reported previously. Furthermore, we observed that SSF2 effectively corrected coronary artery and stent artifacts in patients with a higher heart rate (>85 bpm). When comparing SSF2 with SSF and STD, the correction effect on coronary artery images was higher in both high and low heart rate groups. Additionally, the systolic and diastolic stent inner diameter values of the LM, LAD, LCX, and RCA in the SSF2 group showed increase compared to the SSF and STND groups. This is because motion artifacts will cause the stent lumen to be unclear and the stent inner diameter will be reduced. SSF2 can correct motion artifacts, so the stent inner diameter will increase and be more accurate when SSF2 is used. However, since we did not obtain the true stent diameter, we cannot further determine the impact of SSF2 on the image quality of coronary stents. This is also a limitation. CCTA scan combined with SSF2 algorithm has very important clinical value for patients with coronary heart disease and ISR. Our study provides theoretical support for the early diagnosis and efficacy evaluation of coronary artery disease by SSF2 combined with CCTA.

A previous study has showed that only heart rates that are both high and variable deteriorate image quality.^18^ Our study found that when using the three algorithms, HRV basically has no significant effect on stent image quality. This may be due to the improvement of the model of CT, and 256‐row detector CT scanner has improved the time resolution of imaging.

Additionally, we discovered that the image quality of the coronary arteries and stents deteriorated as the heart rate increased in patients with a heart rate >85 bpm. This may be attributed to the high heart rate shortening the relatively stable phase of the coronary artery, making it more susceptible to motion artifacts. Even though the SSF2 algorithm significantly improves the image quality of CCTA coronary stents, there are still some metal‐induced artifacts that affect the image quality inside and outside the stent lumen, which is a problem to be solved.

In summary, our research confirmed that SSF2 can improve the subjective and objective scores of patients' CCTA images, indicating that SSF2 can significantly correct for motion artifacts in coronary arteries, stents of coronary arteries, myocardium, valves, interventricular septum, atrial septum, and even artificial valves. This helps doctors accurately measure coronary artery stenosis and coronary stent stenosis in patients and improves the accuracy of doctors' diagnosis of coronary artery stenosis, in‐stent restenosis and prosthetic valve abnormalities.

Due to the capacity of SSF2 to enhance image quality in patients with elevated heart rates, SSF2 may be particularly helpful for individuals contraindicated or intolerant to beta‐blockers, thereby facilitating CCTA imaging in patients with contraindications or intolerance to heart rate‐lowering medications.

However, there are several limitations in this study that should be acknowledged. Firstly, the study primarily focuses on subjective and objective evaluations of image quality, with limited inclusion of patients with ISR. Furthermore, invasive coronary angiography was not employed to validate the accuracy of SSF2 in diagnosing ISR lesions, necessitating further research in the future. Secondly, the stent models used for each patient were not considered, resulting in non‐uniformity in the metallic composition and imprecise definition of long and inner diameters, which were roughly estimated at 3 mm. Thirdly, the sample size of patients with stents and high heart rates was small, potentially impacting the generalizability of the findings. Fourthly, this study was conducted at a single center, making it imperative to conduct multi‐center studies with large sample sizes to verify and support the value of SSF2 technology in CCTA inspections and its potential for future implementation. Moreover, we did not compare diastolic and systolic image quality horizontally, which can be further studied in future experiments. An additionally limitation was that only had two readers were used. Lastly, we solely evaluated the differences between various reconstruction algorithms of the same CT scanner, but did not compare them with other imaging techniques such as dual‐source CT imaging.

## 5 CONCLUSIONS

Compared to traditional SSF images and standard images, SSF2 offers significant improvements in both the subjective and objective scores when visualizing coronary arteries, myocardium, MV, TV, AV, PV, VS, AS, PA, and coronary stents. Moreover, SSF2 has shown notable advancements in image quality specifically for patients with a heart rate equal to or exceeding 85 bpm. HRV basically has no significant effect on stent image quality with any of the three algorithms.

## AUTHOR CONTRIBUTIONS

**Zhehao Wu**: Collected data; conducted statistical analysis; prepared the original manuscript draft. **Qijia Han**: Provided essential theoretical insights. **Zhijuan Zheng**: Assisted with statistical analysis. **Zhu Ai**: Provided essential theoretical insights. **Yuying Liang**: Provided essential theoretical insights. **Minyi Wu**: Provided essential theoretical insights. **Kun Ma**: Assisted with manuscript writing and revisions. **Zhiming Xiang**: Conceptualized and supervised the project.

## CONFLICT OF INTEREST STATEMENT

All the authors declare no competing interests. There are no disclosures relevant to the subject matter of this article.

## ETHICAL APPROVAL

Institutional Review Board approval was obtained (PYRC‐2023‐330).

## INFORMED CONSENT

Oral informed consent was waived by the Institutional Review Board.

## Supporting information

Supporting Information

Supporting Information

Supporting Information

## Data Availability

The data that support the findings of this study are available from the corresponding author upon reasonable request.
