# Supplementary material for: Enhancing diagnostic performance and image quality in coronary CT angiography: Impact of SnapShot Freeze 2 algorithm across varied heart rates in stent patients
Source: J Appl Clin Med Phys. 2024 May 28;25(8):e14412. doi: 10.1002/acm2.14412 (PMC11302822; doi:10.1002/acm2.14412)
Supplement: Supplementary file 1 — Supporting Information [file ACM2-25-e14412-s003.docx]

| **Supplementary Table 1**  Objective Assessment of Arteries、Myocardium and MV, TV, AV, PV. | | | | | |
| --- | --- | --- | --- | --- | --- |
| Parameters | Locations | Period | p(SSF2 v SSF) | p(SSF2 v STD) | p(SSF v STD) |
| SNR  n=118 | AO | Systole | p＜0.001 | 0.01 | 0.002 |
|  |  | Diastole | p＜0.001 | p＜0.001 | 0.009 |
|  |  |  |  |  |  |
|  | LCA | Systole | p＜0.001 | p＜0.001 | 0.816 |
|  |  | Diastole | p＜0.001 | p＜0.001 | 0.034 |
|  |  |  |  |  |  |
|  | LAD | Systole | p＜0.001 | p＜0.001 | 0.262 |
|  |  | Diastole | p＜0.001 | p＜0.001 | 0.241 |
|  |  |  |  |  |  |
|  | RCA | Systole | p＜0.001 | p＜0.001 | 0.082 |
|  |  | Diastole | p＜0.001 | p＜0.001 | 0.678 |
|  |  |  |  |  |  |
|  | Myocardium | Systole | p＜0.001 | 0.126 | p＜0.001 |
|  |  | Diastole | p＜0.001 | 0.001 | 0.017 |
|  |  |  |  |  |  |
|  | mitral valve | Systole | p＜0.001 | p＜0.001 | p＜0.001 |
|  |  | Diastole | p＜0.001 | 0.001 | p＜0.001 |
|  |  |  |  |  |  |
|  | tricuspid valve | Systole | p＜0.001 | 0.001 | 0.019 |
|  |  | Diastole | p＜0.001 | 0.003 | 0.062 |
|  |  |  |  |  |  |
|  | pulmonary valve | Systole | p＜0.001 | 0.01 | 0.001 |
|  |  | Diastole | p＜0.001 | 0.047 | 0.004 |
|  |  |  |  |  |  |
|  | aortic valve | Systole | p＜0.001 | 0.039 | p＜0.001 |
|  |  | Diastole | p＜0.001 | 0.001 | p＜0.001 |
|  |  |  |  |  |  |
| CNR  n=118 | AO | Systole | p＜0.001 | 0.165 | 0.001 |
|  |  | Diastole | 0.002 | 0.338 | p＜0.001 |
|  |  |  |  |  |  |
|  | LCA | Systole | p＜0.001 | p＜0.001 | p＜0.001 |
|  |  | Diastole | p＜0.001 | p＜0.001 | 0.313 |
|  |  |  |  |  |  |
|  | LAD | Systole | p＜0.001 | 0.001 | 0.932 |
|  |  | Diastole | 0.001 | 0.027 | 0.176 |
|  |  |  |  |  |  |
|  | RCA | Systole | p＜0.001 | 0.024 | 0.016 |
|  |  | Diastole | p＜0.001 | 0.076 | 0.002 |
|  |  |  |  |  |  |
|  | Myocardium | Systole | 0.016 | 0.641 | 0.003 |
|  |  | Diastole | 0.003 | 0.285 | 0.001 |
|  |  |  |  |  |  |
|  | mitral valve | Systole | p＜0.001 | 0.11 | 0.001 |
|  |  | Diastole | 0.002 | 0.281 | p＜0.001 |
|  |  |  |  |  |  |
|  | tricuspid valve | Systole | p＜0.001 | 0.079 | 0.001 |
|  |  | Diastole | 0.004 | 0.1 | 0.001 |
|  |  |  |  |  |  |
|  | pulmonary valve | Systole | 0.002 | 0.177 | 0.002 |
|  |  | Diastole | 0.003 | 0.212 | 0.001 |
|  |  |  |  |  |  |
|  | aortic valve | Systole | p＜0.001 | 0.124 | 0.001 |
|  |  | Diastole | 0.002 | 0.292 | 0.001 |
|  |  |  |  |  |  |
| Note. The difference was statistically significant when P < 0.0167. SNR: signal-to-noise ratio; CNR: contrast to noise ratio; LAD: left anterior descending artery; RCA: right coronary artery; AO: aorta; LCA: left coronary artery. | | | | | |

| **Supplementary Table 2** Objective Assessment of Stents | | | | | |
| --- | --- | --- | --- | --- | --- |
| Parameter | Location | Period | SSF2 v SSF | SSF2 v STND | SSF v STND |
| AI | LAD Stent n=89 | Systole | p＜0.001 | p＜0.001 | 0.006 |
|  |  | Diastole | p＜0.001 | p＜0.001 | 0.699 |
|  |  |  |  |  |  |
|  | LCX Stent n=35 | Systole | p＜0.001 | p＜0.001 | 0.136 |
|  |  | Diastole | p＜0.001 | p＜0.001 | 0.287 |
|  |  |  |  |  |  |
|  | RCA Stent n=46 | Systole | 0.016 | p＜0.001 | 0.015 |
|  |  | Diastole | 0.001 | 0.001 | 0.658 |
|  |  |  |  |  |  |
|  | LM Stent n=7 | Systole | 0.018 | 0.028 | 0.31 |
|  |  | Diastole | 0.028 | 0.018 | 0.866 |
|  |  |  |  |  |  |
| CNR of Stents | LAD Stent n=89 | Systole | p＜0.001 | p＜0.001 | 0.019 |
|  |  | Diastole | p＜0.001 | p＜0.001 | 0.745 |
|  |  |  |  |  |  |
|  | LCX Stent n=35 | Systole | p＜0.001 | p＜0.001 | 0.219 |
|  |  | Diastole | p＜0.001 | p＜0.001 | 0.213 |
|  |  |  |  |  |  |
|  | RCA Stent n=46 | Systole | 0.001 | p＜0.001 | 0.12 |
|  |  | Diastole | p＜0.001 | 0.001 | 0.537 |
|  |  |  |  |  |  |
|  | LM Stent n=7 | Systole | 0.018 | 0.028 | 0.398 |
|  |  | Diastole | 0.063 | 0.018 | 0.237 |
|  |  |  |  |  |  |
| Inner Diameters | LAD Stent n=89 | Systole | p＜0.001 | p＜0.001 | 0.236 |
|  |  | Diastole | p＜0.001 | p＜0.001 | 0.093 |
|  |  |  |  |  |  |
|  | LCX Stent n=35 | Systole | 0.073 | 0.006 | 0.03 |
|  |  | Diastole | 0.14 | 0.066 | 0.83 |
|  |  |  |  |  |  |
|  | RCA Stent n=46 | Systole | 0.036 | p＜0.001 | 0.005 |
|  |  | Diastole | 0.197 | 0.005 | 0.042 |
|  |  |  |  |  |  |
|  | LM Stent n=7 | Systole | 0.157 | 0.131 | 1 |
|  |  | Diastole | 0.461 | 0.686 | 0.713 |
|  |  |  |  |  |  |
| Note. The difference was statistically significant when P < 0.0167. AI: Artifact index; CNR: Contrast to noise ratio; LAD: left anterior descending artery; LCX: Left circumflex branch; RCA: right coronary artery; LM: left main artery. | | | | | |

| **Supplementary Table 3** Correlation between subjective score of stents and heart rate variability | | | |
| --- | --- | --- | --- |
| Locations | Parameters | P value | |
|  |  |  |  |
| LAD Stent | SSF2 systole | 0.891 |  |
|  | SSF systole | 0.459 |  |
|  | STND systole | 0.87 |  |
|  | SSF2 diastole | 0.536 |  |
|  | SSF diastole | 0.541 |  |
|  | STND diastole | 0.44 |  |
| LCX Stent | SSF2 systole | 0.848 |  |
|  | SSF systole | 0.785 |  |
|  | STND systole | 0.91 |  |
|  | SSF2 diastole | 0.104 |  |
|  | SSF diastole | 0.81 |  |
|  | STND diastole | 0.292 |  |
| RCA Stent | SSF2 systole | 0.979 |  |
|  | SSF systole | 0.54 |  |
|  | STND systole | 0.718 |  |
|  | SSF2 diastole | 0.79 |  |
|  | SSF diastole | 0.759 |  |
|  | STND diastole | 0.033 |  |
| Note. The asterisks * represent statistical significance levels of P < 0.05. LAD: left anterior descending artery; LCX: Left circumflex branch; RCA: right coronary artery. | | | |
|  |  |  |  |
|  |  |  |  |

| **Supplementary Table 4**  Objective Assessment of Arteries | | | | | |
| --- | --- | --- | --- | --- | --- |
| Locations | Parameters | SSF2 Growth rate | | SSF Growth rate | |
|  |  | ≥85 | ＜85 | ≥85 | ＜85 |
| AO | SNR S | 11.70% | 0.60% | -5.80% | -0.70% |
|  | SNR D | 4.50% | 1.20% | 2.80% | -0.20% |
|  | CNR S | 6% | 1.10% | -0.80% | -1.20% |
|  | CNR D | 6.50% | -0.30% | -5.80% | -2% |
| LCA | SNR S | 20.90% | 7.40% | 2.50% | -1.20% |
|  | SNR D | 30.70% | 10% | 0.10% | -1.40% |
|  | CNR S | 6% | 2.70% | -2.10% | -2.50% |
|  | CNR D | 7.20% | 5.60% | -1.10% | 0.80% |
| LAD | SNR S | 40.20% | 13.10% | 4.80% | -0.70% |
|  | SNR D | 25.10% | 10% | 5.70% | -0.40% |
|  | CNR S | 25.70% | 4.10% | 0.90% | 0.40% |
|  | CNR D | 15.80% | 0.40% | -2.50% | -1.50% |
| RCA | SNR S | 14.50% | 15.40% | 2.30% | 5.30% |
|  | SNR D | 23% | 17.90% | 3.60% | -2.70% |
|  | CNR S | 5.90% | 1.90% | -1.10% | -1.30% |
|  | CNR D | 11.70% | 1.50% | -5.80% | -1.60% |
| Mean | | 15.70% | 5.80% | -0.10% | -0.70% |
| Note. SNR: signal-to-noise ratio ; CNR: Contrast to noise ratio; LAD: left anterior descending artery; RCA: right coronary artery; AO:aorta; LCA: left coronary artery. | | | | | |

| **Supplementary Table 5** Objective Assessment of Stents | | | | | |
| --- | --- | --- | --- | --- | --- |
| Locations | Parameters | SSF2 Improvement | | SSF Improvement | |
|  |  | ≥85 | ＜85 | ≥85 | ＜85 |
| LAD Stent | LAD AI S | 26.70% | 26.80% | 9.70% | 3.70% |
|  | LAD AI D | 27.80% | 17.10% | 4.20% | 0.20% |
|  | LAD CNR S | 19.80% | 16.60% | 10.80% | 1.50% |
|  | LAD CNR D | 14.30% | 7.40% | 1% | 0.10% |
|  | LAD DIA S | 23.70% | 11.20% | -0.50% | 2.10% |
|  | LAD DIA D | 21.90% | 7% | 3.60% | 2.50% |
| LCX Stent | LCX AI S | 35.30% | 30.30% | -1.90% | 12.50% |
|  | LCX AI D | 31.70% | 18.30% | 4.70% | 2.10% |
|  | LCX CNR S | 25.10% | 28.10% | 0.70% | 9% |
|  | LCX CNR D | 37.40% | 11.30% | 5.50% | 4.90% |
|  | LCX DIA S | 4.40% | 10.40% | 0 | 7.70% |
|  | LCX DIA D | -33.60% | 1.20% | -21.40% | 0.60% |
| RCA Stent | RCA AI S | 33.60% | 23% | 21.40% | 13.10% |
|  | RCA AI D | 26.50% | 20.40% | -33.90% | 9.30% |
|  | RCA CNR S | 25.40% | 15.90% | 9.30% | 8% |
|  | RCA CNR D | 21.60% | 14% | 1.80% | 2.20% |
|  | RCA DIA S | 15.40% | 10.10% | 14.70% | 5.60% |
|  | RCA DIA D | 33.30% | 7.90% | 17% | 6.90% |
| LM Stent | LM AI S | 33.50% | 34% | 3.50% | 3.20% |
|  | LM AI D | 39.90% | 21.50% | 7.10% | 1.80% |
|  | LM CNR S | 15.80% | 34.50% | 4.10% | 0.90% |
|  | LM CNR D | 63.80% | 18.20% | 21.10% | 4.80% |
|  | LM DIA S | 3.20% | 4.30% | 1.60% | -1.40% |
|  | LM DIA D | -6.30% | 16.40% | -9.50% | 3.30% |
| Mean | | 22.50% | 16.90% | 3.10% | 4.40% |
| Note. AI :Artifact index ; CNR:Contrast to noise ratio;LAD:left anterior descending artery;LCX:Left circumflex branch;RCA:right coronary artery;LM:left main artery. | | | | | |
|  |  |  |  |  |  |
|  |  |  |  |  |  |

| **Supplementary Table 6**  Objective Assessment of Valves | | | | | |
| --- | --- | --- | --- | --- | --- |
| Locations | Parameters | SSF2 Growth rate | | SSF Growth rate | |
|  |  | ≥85 | ＜85 | ≥85 | ＜85 |
| mitral valve | SNR S | 5.70% | 5.80% | -1% | -1% |
|  | SNR D | 5.70% | 3.10% | -1% | -0.90% |
|  | CNR S | 5.90% | 18% | -0.80% | -1.20% |
|  | CNR D | 6.70% | 0.02% | -5.90% | -2% |
| tricuspid valve | SNR S | 8.10% | -3% | -0.10% | -1.10% |
|  | SNR D | 3.80% | 1.60% | -1.60% | -0.40% |
|  | CNR S | 8.50% | 1.30% | 1% | -1.10% |
|  | CNR D | 4.30% | 1.40% | -6.90% | -1.40% |
| pulmonary valve | SNR S | 1.40% | 0.10% | -0.60% | -1.20% |
|  | SNR D | 3.70% | 1% | -1.20% | 0% |
|  | CNR S | 6.60% | 0.40% | 0.40% | -1.10% |
|  | CNR D | 4.40% | 0.70% | -6.60% | -1.30% |
| aortic valve | SNR S | 4.20% | 1.50% | -2.20% | -4.10% |
|  | SNR D | 2.90% | 2.30% | -2.10% | -1% |
|  | CNR S | 6.40% | 1.20% | -0.80% | -1.80% |
|  | CNR D | 5.80% | 0.40% | -5.90% | -1.40% |
| Mean | | 5.30% | 2.70% | -2.20% | -1.30% |
| Note. SNR: signal-to-noise ratio ; CNR: Contrast to noise ratio; | | | | | |

| **Supplementary Table 7** Objective Assessment of Myocardium | | | | | |
| --- | --- | --- | --- | --- | --- |
| Locations | Parameters | SSF2 Growth rate | | SSF Growth rate | |
|  |  | ≥85 | ＜85 | ≥85 | ＜85 |
| myocardial | SNR S | 5.10% | 0.90% | -1.70% | -3.50% |
|  | SNR D | 2.90% | 3.60% | -2.40% | -0.10% |
|  | CNR S | 5% | 0% | -0.80% | -1.40% |
|  | CNR D | 4.60% | 0% | -5.40% | -2.20% |
| Mean | | 4.40% | 1.10% | -2.60% | -1.80% |
| Note. SNR: signal-to-noise ratio ; CNR: Contrast to noise ratio. | | | | | |

| **Supplementary Table 8**  Subjective Assessment of Arteries | | | | |
| --- | --- | --- | --- | --- |
| Locations | SSF2 Improvement | | SSF Improvement | |
|  | ≥85 | ＜85 | ≥85 | ＜85 |
| AO S | 18.20% | 6.10% | 5.50% | 1.50% |
| AO D | 15.70% | 4.40% | 0.90% | 0.50% |
| LCA S | 12.30% | 6.60% | 5.30% | 1.30% |
| LCA D | 13.60% | 3.50% | 2.70% | 1.20% |
| LAD S | 20% | 8.30% | 7% | 1.60% |
| LAD D | 12.30% | 3.50% | 3.80% | 1.50% |
| RCA S | 19.40% | 12.10% | 12% | 8.40% |
| RCA D | 18.40% | 8.70% | 8.70% | 2.30% |
| Mean | 16.20% | 6.70% | 5.70% | 2.30% |
| Note. LAD: left anterior descending artery; RCA: right coronary artery; AO:aorta; LCA: left coronary artery; S:Systole; D:Diastole.. | | | | |

| | **Supplementary Table 9**  Subjective Assessment of Stents | | | | | | --- | --- | --- | --- | --- | | Locations | SSF2 Improvement | | SSF Improvement | | | ≥85 | ＜85 | ≥85 | ＜85 | | LM Stents S | 40% | 33.30% | 0% | 11.10% | | LM Stents D | 62.50% | 0% | 12.50% | 0% | | LAD Stents S | 39.50% | 32.40% | 18.40% | 9.40% | | LAD Stents D | 39.50% | 23.60% | 10.50% | 11.40% | | LCX Stents S | 26.30% | 25.60% | 10.50% | 14% | | LCX StentsD | 42.10% | 18.40% | 5.30% | 18.40% | | RCA Stents S | 46.40% | 28.10% | 32.10% | 23.40% | | RCA Stents D | 45.20% | 24.60% | 22.60% | 18.50% | | Mean | 42.70% | 23.30% | 14% | 13.30% | | Note. LAD: left anterior descending artery; LCX: Left circumflex branch; RCA: right coronary artery; LM: left main artery; S:Systole; D:Diastole.. | | | | |  | **Supplementary Table 10** Subjective Assessment of AV, PV, MV, TV | | | | | | --- | --- | --- | --- | --- | | Locations | SSF2 Improvement | | SSF Improvement | | | ≥85 | ＜85 | ≥85 | ＜85 | | MV S | 5% | 3.30% | 0% | 0% | | MV D | 3.90% | 1.40% | 0% | 0.30% | | TV S | 0% | 0.60% | 0% | 0% | | TV D | 0% | 0.90% | 0% | 0.30% | | AV S | 3.80% | 2.80% | 0% | 0% | | AV D | 4.90% | 3.10% | 0% | 0% | | PV S | 0.90% | 0.60% | 0% | 0% | | PV D | 2.90% | 1.40% | 0% | 0% | | mean | 2.70% | 1.80% | 0% | 0.10% | | MV: mitral valve; TV: tricuspid valve; AV: aorta valve; PV: pulmonary valve; S:Systole; D:Diastole. | | | | |  | **Supplementary Table 11**  Subjective Assessment of AO, PA, VS, AS | | | | | | --- | --- | --- | --- | --- | | Locations | SSF2 Improvement | | SSF Improvement | | | ≥85 | ＜85 | ≥85 | ＜85 | | AO S | 2.90% | 1.40% | 0% | 0% | | AO D | 6.90% | 0.80% | 0% | 0% | | PA S | 1% | 0.80% | 0% | 0% | | PA D | 2.90% | 0.60% | 0% | 0.30% | | VS S | 1% | 1.70% | 0% | 0% | | VS D | 3.90% | 0.30% | 0% | 0% | | AS S | 3.90% | 2.60% | 0% | 0% | | AS D | 6.10% | 2.70% | 0% | 0% | | mean | 3.60% | 1.40% | 0% | 0% | | AO: aortic root; PA: pulmonary artery root; AS: atrial septum; VS: ventricular septumS:Systole; D:Diastole.. | | | | | |
| --- | --- | --- | --- | --- | --- | --- | --- | --- | --- | --- | --- | --- | --- | --- | --- | --- | --- | --- | --- | --- | --- | --- | --- | --- | --- | --- | --- | --- | --- | --- | --- | --- | --- | --- | --- | --- | --- | --- | --- | --- | --- | --- | --- | --- | --- | --- | --- | --- | --- | --- | --- | --- | --- | --- | --- | --- | --- | --- | --- | --- | --- | --- | --- | --- | --- | --- | --- | --- | --- | --- | --- | --- | --- | --- | --- | --- | --- | --- | --- | --- | --- | --- | --- | --- | --- | --- | --- | --- | --- | --- | --- | --- | --- | --- | --- | --- | --- | --- | --- | --- | --- | --- | --- | --- | --- | --- | --- | --- | --- | --- | --- | --- | --- | --- | --- | --- | --- | --- | --- | --- | --- | --- | --- | --- | --- | --- | --- | --- | --- | --- | --- | --- | --- | --- | --- | --- | --- | --- | --- | --- | --- | --- | --- | --- | --- | --- | --- | --- | --- | --- | --- | --- | --- | --- | --- | --- | --- | --- | --- | --- | --- | --- | --- | --- | --- | --- | --- | --- | --- | --- | --- | --- | --- | --- | --- | --- | --- | --- | --- | --- | --- | --- | --- | --- | --- | --- | --- | --- | --- | --- | --- | --- |
