# Supplementary material for: Enhancing diagnostic performance and image quality in coronary CT angiography: Impact of SnapShot Freeze 2 algorithm across varied heart rates in stent patients
Source: J Appl Clin Med Phys. 2024 May 28;25(8):e14412. doi: 10.1002/acm2.14412 (PMC11302822; doi:10.1002/acm2.14412)
Supplement: Supplementary file 2 — Supporting Information [file ACM2-25-e14412-s002.docx]

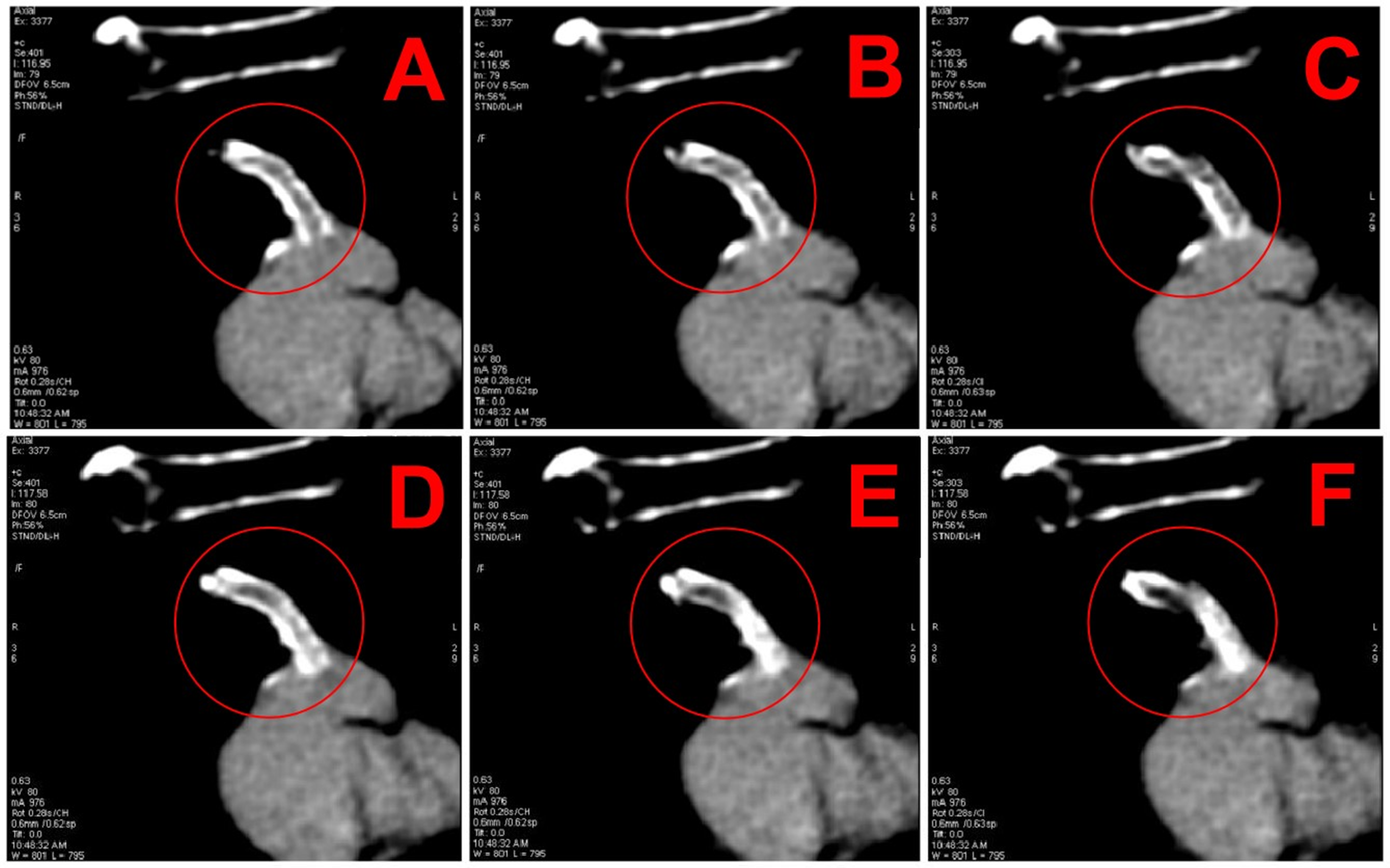


- **Supplementary Figure 1** A 70-year-old female with RCA stent.The quality of the image reconstructed with SSF2 is excellent and there is no motion artifact. The electrocardiogram report showed that the HR was 123 beats/min during the scan. Assessment of Atrial RCA stent: A, D: SSF2 images , (score 1). B, E: SSF images, (score 2) . C, F: STND images, (score 3).
