# Supplementary material for: Enhancing diagnostic performance and image quality in coronary CT angiography: Impact of SnapShot Freeze 2 algorithm across varied heart rates in stent patients
Source: J Appl Clin Med Phys. 2024 May 28;25(8):e14412. doi: 10.1002/acm2.14412 (PMC11302822; doi:10.1002/acm2.14412)
Supplement: Supplementary file 3 — Supporting Information [file ACM2-25-e14412-s001.docx]

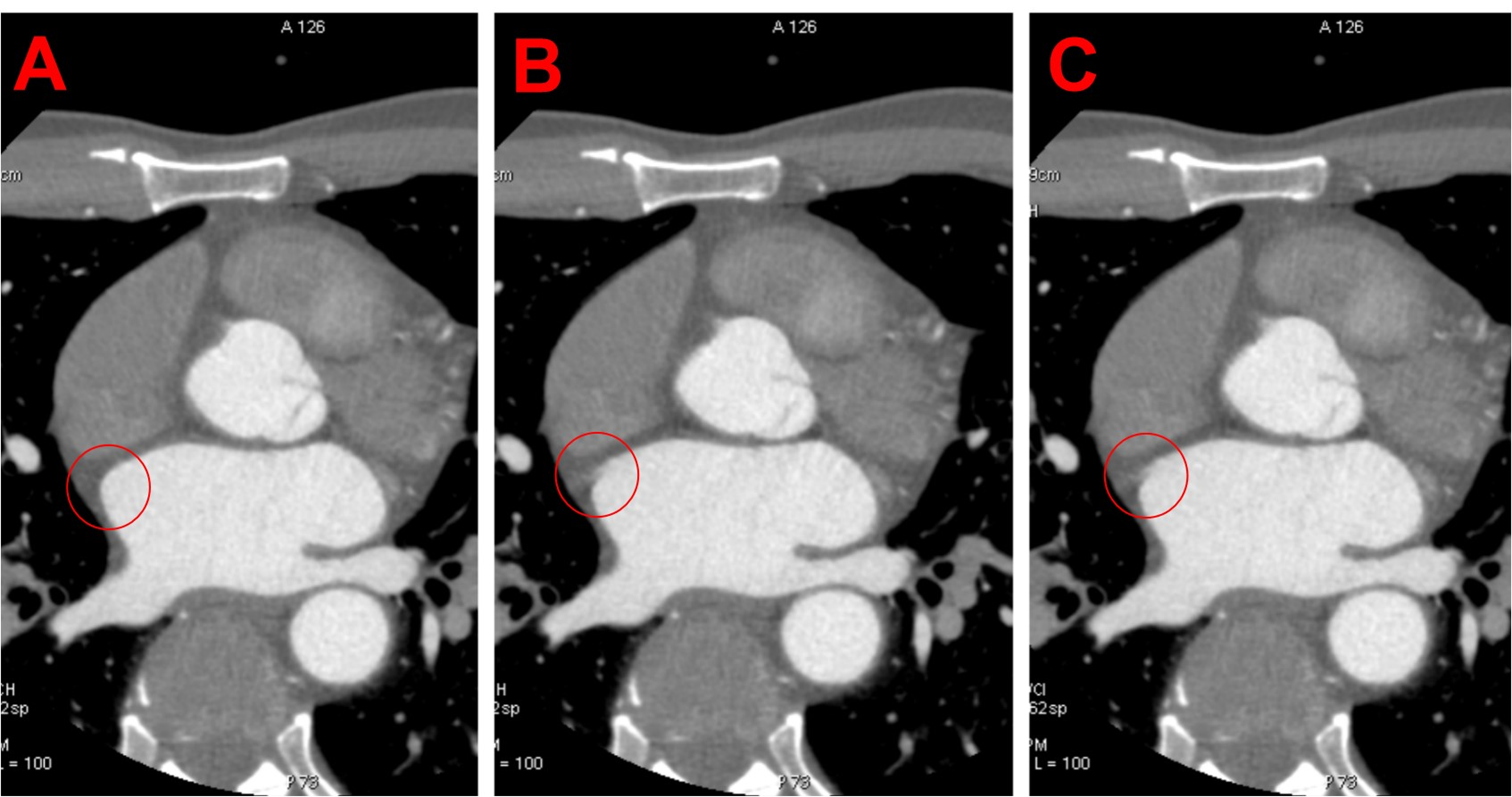


**Supplementary Figure 2** A 60-year-old male. Assessment of Atrial septum. The quality of the image reconstructed with SSF2 is excellent and there is no motion artifact. The electrocardiogram report showed that the HR was 82 beats/min during the scan. Assessment of Atrial septum: A: SSF2 images, (score 4). B: SSF images, (score 3) . C: STND images, (score 3) .
